# Anxiety and coping in women with breast cancer in chemotherapy**^1^**

**DOI:** 10.1590/1518-8345.1722.2891

**Published:** 2017-06-05

**Authors:** Araceli Vicente da Silva, Eliana Zandonade, Maria Helena Costa Amorim

**Affiliations:** 2 MSc, RN, Hospital Santa Rita de Cássia, Vitória, ES, Brazil; 3 PhD, Professor, Departamento de Estatística, Universidade Federal do Espírito Santo, Vitória, ES, Brazil.; 4 PhD, Professor, Departamento de Enfermagem, Universidade Federal do Espírito Santo, Vitória, ES, Brazil.

**Keywords:** Breast Neoplasm, Adaptation Psychological, Chemotherapy, Anxiety

## Abstract

**Objective:**

to identify the coping strategies used by women with breast cancer in chemotherapy and to verify the association with the anxiety profile presented by them.

**Method:**

cross-sectional study of the analytical type. We used a random sample of 307 women with cancer in previous chemotherapy, adjuvant or palliative treatment. The data was collected using an interview technique with form registration, active search in medical records, Scale of Mode of Confronting Problems and Inventory of Anxiety and State. The Statistical Package for Social Sciences 19.0, Pearson correlation coefficient and the test Mann-Whitney were used.

**Results:**

there was a significant association of the anxiety trait and problem-focused coping strategies with a focus on emotion (p<0,000) and the anxiety state with problem-focused coping (p=0,001) and with focus on emotion (p=0,004). The results demonstrate weak associations between different coping strategies.

**Conclusion:**

the coping strategy chosen by women with breast cancer is directly related to anxiety. Patients with low-level anxiety tend to use problem-solving strategies while emotion-focused coping is applied if the level is medium to high.

## Introduction

According to the National Cancer Institute (INCA), breast cancer is the most common type of cancer in women if non-melanoma skin cancer is discounted, as estimated for the 2016/2017 biennium, representing an occurrence of 1010 new cases of breast cancer in the state of Espirito Santo and 140 new cases for the capital, Vitoria^1^. Thus, breast cancer is a public health problem, as well as a factor that generates biopsychosocial changes for these women.

As one of the neoplasms most feared by women, breast cancer when diagnosed causes patients to be permeated with negative feelings, distorting the perception of their self-image and inhibiting their sexuality, generating low self-esteem and lack of self-assessment. In addition, the stigma of mutilation caused by surgery generates stress and it can lead to some difficulties, leading women to adopt different types of coping^2^ ^-^ ^3^. 

Coping strategies consist of constant cognitive and behavioral efforts to manage specific external and / or internal demands arising from stress situations that are assessed as overloading or exceeding personal resources, a dynamic process that does not occur by chance and is subject to evaluation and reassessment. The confrontation may be focused on the problem, when the person seeks resolution of the situation through information about the stressful event and thus evaluates the action that believes to be most effective in resolving the stressful event; or it can be focused on emotion, when the strategies adopted are loaded with emotion resulting from self-defense processes that serve as a shield, avoiding the confrontation with the stressful factor. Thus, individuals can take distance, escape or avoid the problem^4^. The association of coping with cognitive evaluation becomes a mediator between the organism and the environment, making the process dynamic and multidimensional^5^. Coping is considered a strategy, so it can be taught, used and adapted, regardless of the stress situation that the individual is experiencing. If the coping strategy used is efficient, the stress will be overcome. Otherwise, there will be a process of cognitive reassessment of the stressor and possible changes of actions will occur, until the problem is solved or exhaustion^6^. 

Stress can be understood as a process that requires a response, triggered by several steps, that will be evaluated by individuals with the purpose of seeking its meaning, so that the person in the future will be able to choose an adequate coping way in a given situation. Thus, stress is an inevitable fact that is part of the evolutionary cycle of the human being, and each person, when faced with a stressor agent, will seek a confrontation based on their experiences, their values, their feelings and their culture^6^. Stress involves endocrine reactions, emotions, beliefs, culture and mood variation, stressing the relationship of stress with coping strategies, with fear and with anxiety. Anxiety can be linked to a stress response in an unconscious process where the causative agent is not fully understood or defined^7^.

To feel anxious is different from being anxious. Thus, one can divide anxiety into state and trace anxiety. Anxiety, or Anxiety-E, is defined as a transient emotional activity, according to the momentary conditions experienced by the individual. Emphasis is placed on consciously perceived feelings of tension and apprehension, which may vary in intensity. But regarding to the trait of anxiety, or Anxiety-T, is how people usually react to everyday stress situations perceived as threatening, and the trait of anxiety is stable, varying according to each individual. Overall, Anxiety-T levels are directly proportional to Anxiety-E levels, since people who have high levels of Anxiety-T tend to react more frequently to situations as if they are threatening or dangerous, raising levels of Anxiety-E^8^.

Women, when diagnosed with breast cancer and the information that they will have to undergo chemotherapy, are affected by innumerable sensations that generate stress and may make them anxious, which will require to adopt a strategy of coping. The way women will face this peculiar moment in their lives and how they will make decisions is of utmost importance, if they can use of a coping strategy that best fits with that moment. If it is successful, this will allow them to live and overcome this moment in the best way possible, minimizing anxiety and stress. However, if the coping type chosen is not effective, this can be disastrous, which can generate more stress and increase anxiety levels. Given this reality, and considering the experience of the researcher in the chemotherapy sector of a philanthropic institution, where women of all social classes are treated, with different perceptions and diverse experiences, and where every day at least one woman starts chemotherapy treatment, being it previous, adjuvant or palliative to treat breast cancer, and as they do not always adopt an efficient coping, there was a need to verify if the coping strategies experienced by women with breast cancer undergoing chemotherapy were influenced by their anxiety levels.

In view of the above, the present study aims to identify the coping strategies used by women with breast cancer in chemotherapy and to verify the association with the anxiety profile presented by them.

## Method

The research was carried out in the chemotherapy sector of the Ylza Bianco outpatient clinic, which belongs to the Santa Rita de Cássia Hospital (HSRC). Its main activity is the Feminine Association for Education and Fight against Cancer (AFECC), located in the city of Vitoria, Espirito Santo. It is a cross-sectional study of the analytical type.

The study was composed of women aged 18 years and over, diagnosed with breast cancer and who were undergoing previous intravenous chemotherapy, adjuvant or palliative. Patients with any type of psychosis, mental or auditory deficit that might impair the interview with the researcher were excluded from the sample. Such data were identified in the interdisciplinary consultations carried out before the beginning of the cancer treatment and recorded in the patients' medical records. It is worth noting that the number of the chemotherapy cycle that the patient was performing at the time of data collection did not represent an inclusion or exclusion criterion in the research.

The data were collected from March to May 2015, in the chemotherapy sector, during a meeting with the patients while they received the intravenous chemotherapy treatment; These women were invited to participate in the study and then submitted to the Free and Informed Consent Form, which should be signed and a copy delivered to the patient and another copy to the researcher. The sociodemographic variables and the clinical variable participation in the Rehabilitation Program for Mastectomized Women (PREMMA) were collected by means of the interview technique with registration in their own forms, and the other clinical variables were obtained through active search in the medical records. As an instrument to identify the coping strategies adopted by breast cancer patients undergoing chemotherapy, we used the Conflict Mode Scale (EMEP), validated for Portuguese through the factorial analysis of a Brazilian population composed of people of the population in general and by people affected by chronic diseases^5^. EMEP is composed of 45 items that are distributed in four factors: 18 items related to the problem-focused coping; 15 items related to emotion-focused coping; seven items related to the religious search and five items referring to the search for social support. To answer EMEP questions, the five-point Likert scale was used, where one equals "I never do this" and five equals "I always do this". To analyze the patients' responses, scores ranging from one to five were used, and the highest ones indicated that a particular form of coping was being used more frequently. Trait and anxiety status were measured using the instrument *STAI- State Trait Anxiety Inventory* ^8^, known in Brazil as Anxiety (A-Trait) e Estado (B-State) Inventory (IDATE), translated and validated to Portuguese in 1979^9^. This instrument contains 20 questions dedicated to the analysis of the anxiety trait of the women studied and 20 questions to analyze the state of anxiety at the time of the interview. The frequency of the anxiety trait ranges from one to four, one = almost never, two = sometimes, three = frequently and four = almost always. The score for the frequency of the anxiety state also varies from one to four, where one = no, two = a little, three = a lot and four = totally. The score of these items varies from 20 to 80 points, with 20 to 39 points indicating low anxiety level, 40 to 59 points mean level of anxiety and 60 to 80 points high level of anxiety.

For statistical analysis we used SPSS - Statistical Package for Social Sciences - version 19.0. The Pearson correlation coefficient was used, the Mann-Whitney test and a significance level of 5% was set corresponding to p = 0,05 (confidence limit of 95%). The calculation was performed in the Epidat program, version 4.0, to estimate the correlation between anxiety and coping; assuming a minimum correlation of 0.200, that is, at least weak; a significance level of 5% and test power of 95%. The minimum sample size calculated was 266 patients. The researcher performed a sampling plan, in random days and times, contemplating every day of the week and all shifts, morning and afternoon. The collection was done addressing the women who received chemotherapy in these days and times, arriving at a sample of 307 patients.

The study was submitted to the Research Ethics Committee of the Health Sciences Center of the Federal University of Espirito Santo (CCS / UFES) within the ethical regulation foreseen in the resolution nº 466/12. After evaluation it was in approved on March 11, 2015 under the nº 980.091. 

## Results 

We interviewed 307 women, of which 36.5% had between 41 and 50 years, 54.7% had a partner, 52.1% had from one to eight years of study, 49.8% were evangelical, 60.3% Lived in the metropolitan region, 65% belonged to economic class C1 and C2, and 46.3% reported having family income between one and two minimum wages. Regarding the clinical variables, 37.1% had staging IV, 52.4% were receiving adjuvant chemotherapy and 51.5% were performing the first, second or third cycle of chemotherapy, with AC (doxorubicin and cyclophosphamide) the most used chemotherapy protocol (33.9%). Regarding their participation in PREMMA, 87.9% said they had never participated of the group. 

Table 1 shows the anxiety trait and coping strategies used by breast cancer patients under treatment with intravenous chemotherapy.

It was verified (Table 1) that patients undergoing chemotherapy treatment who used as a form of coping the focus on the problem (median: 3.89) and the focus on social support (median: 3.00) showed to have low level of trait of anxiety. However, patients who use as a coping strategy the focus on emotion (median: 2.00) or focus on religion (median: 3.86) have medium to high levels of anxiety trait. Only the association of the anxiety trait with the problem-focused coping strategies (p <0.000) and with a focus on emotion (p <0.000) was significant.

**Table 1:** Trait anxiety and coping strategies used by women with breast cancer undergoing chemotherapy. Ylza Bianco Outpatient Clinic - HSRC/AFECC. Vitória, ES, Brazil, 2015

Variable	Anxiety Trait							p-value
	Low level of anxiety				Medium and high level of anxiety			
	Mean	SD*	Medium		Mean	SD*	Medium	
Focus on problem	3,86	0,39	3,89		3,61	0,47	3,61	0,000
Focus on emotion	1,67	0,39	1,67		2,07	0,45	2,00	0,000
Focus on religion	3,76	0,56	3,71		3,84	0,54	3,86	0,212
Focus on social support	2,92	0,87	3,00		2,87	0,75	2,80	0,606

*Standard deviation

Regarding the anxiety state (Table 2), the patients who used the focus on the problem (median: 3.83) and the focus on social support (median: 3.00) presented a low level of anxiety as a form of coping. On the other hand, the patients who demonstrated that they had medium to high levels of anxiety at the time of the interview were those who used the emotion-focused coping strategy (median: 1.97) and with a focus on religion (median: 3.86). There was a significant association between the anxiety state and the focus on the problem (p = 0.001), with a focus on emotion (p = 0.004). 

**Table 2:** Anxiety status and coping strategies used by women with breast cancer undergoing chemotherapy. Ylza Bianco Outpatient clinic - HSRC/AFECC. Vitoria, ES, Brazil, 2015

Variables	Anxiety State							p-value
	Low level of anxiety				Medium and High level of anxiety			
	Mean	SD*	Median		Mean	SD*	Median	
Focus on problem	3,79	0,42	3,83		3,50	0,52	3,42	0,001
Focus on emotion	1,80	0,44	1,80		2,15	0,59	1,97	0,004
Focus on religion	3,79	0,54	3,71		3,77	0,69	3,86	0,777
Focus on social support	2,90	0,82	2,80		2,88	0,85	3,00	0,990

*Standard Deviation

When using Pearson's correlation coefficient (Table 3), there are weak but statistically significant correlations. Thus, we observed that the correlation of religious-based coping with coping with focus on emotion (r = 0,136; p = 0,017) and the social-focus coping with the emotion-focused coping (r = 0,123; p = 0,031) have small coefficients. On the other hand, the focus on religion with the focus on the problem (r = 0,329; p < 0,000) and the focus on social support with problem-focused coping (r = 0,349; p < 0,000) demonstrate a reasonable degree of correlation. The correlation of emotion-focused coping strategy with problem-focused coping and coping with social-focus coping with religious focus was not found to be statistically significant.

**Table 3:** Coefficient of correlation of coping strategies and the state and anxiety traits of women with breast cancer undergoing chemotherapy. Ylza Bianco Outpatient clinic - HSRC/AFECC. Vitória, ES, Brazil, 2015

Variable	Statistic	Focus on problem	Focus on emotion	Focus on religion	Focus on social support	Anxiety Trait	Anxiety State
Focus on problem	Pearson	1					
	p-value						
Focus on emotion	Pearson	-0,110	1				
	p-value	0,054					
Focus on religion	Pearson	0,329	0,136	1			
	p-value	0,000	0,017				
Focus on social support	Pearson	0,349	0,123	0,074	1		
	p-value	0,000	0,031	0,196			
Trait	Pearson	-0,297	0,511	0,073	0,014	1	
	p-value	0,000	0,000	0,204	0,801		
State	Pearson	-0,298	0,267	-0,075	-0,018	0,403	1
	p-value	0,000	0,000	0,191	0,749	0,000	

Regarding the anxiety trait, it showed a relation inversely proportional to the focus on the problem (r = -0.297; p <0.000) and a directly proportional relation to the focus on emotion (r = 0.0511; p <0.000). There was no statistically significant correlation between the anxiety trait and the focus on religion and focus on social support (Table 3). The state of anxiety also presented an inverse relation with the confrontation with the focus on the problem (r = -0,298; p < 0,000) and directly proportional relationship with the emotion-focused strategy (r = 0,267; p < 0,000) and with the anxiety trait (r = 0,403; p < 0,000). There was no statistically significant correlation between anxiety state and religion-centered coping with a focus on social support (Table 3).

## Discussion

Any adult in the productive phase, when experiencing a disease, will develop feelings and reactions to this new reality, such as anxiety, fear, anger, denial and insecurity. In addition, this new experience also involves how self-image visualization will take place and how to finance its survival. Faced with this, it becomes important to know the forms of coping adopted by patients and help them redirect their coping, if necessary, to reduce stress and anxiety. After all, the patient will have to deal with potentially stressful moments and experience them in one way or another^7^.

On the research^10^ ^)^ on the influence of anxiety on coping strategies used in the preoperative period, there was a significant anxiety trait and emotion-focused coping strategy (detachment), demonstrating that patients who use this form of coping experience show a mid-level anxiety trait, which is in agreement with the present study that showed that patients with breast cancer in chemotherapy treatment that use as a form of coping the focus on the emotion present medium to high level of anxiety. In contrast, women who use the strategy to focus on problems have a low level of anxiety. Authors^10^ say that by using a strategy with a focus on emotion, patients do not take a posture to eliminate the problem but rather to distance themselves from them, assuming a more defensive posture. Attitudes to avoid the problem foreshadow difficulties for the patient to adjust to the new reality. On the other hand, those who focus on the problem and seek information tend to present better adjustment^11^. The use of emotionally focused coping for breast cancer patients may represent a poor fit since it provides for physical and psychological distress during chemotherapy^12^. A study^13^ conducted with African-American women with breast cancer has shown that the greater the coping capacity the less psychological suffering and negative religious coping.

The patients with breast cancer undergoing chemotherapy that presented low level of anxiety state were those that used, as a form of coping, the focus on the problem. On the other hand, the patients with medium to high level of anxiety were those that used the focus on emotion as a coping strategy, which is in agreement with the results obtained by other authors^10^.

There are factors that will be crucial in choosing the type of strategy to be used, such as the nature of the stressor, the occasions in which it occurs or is repeated, how the person faced the stressor agent in the past, and the style that determines the subject. In this sense, there are people who tend to avoid or minimize the problem and those who face them or are vigilant. Each type of person will develop different reactions that can be constructive, if the individual uses coping mechanisms so that the action occurs in a conscious way, or the action may be defensive, in which case forces originated in their unconscious will determine their behavior^14^. 

In a study about the coping strategies experienced by women with breast cancer using tamoxifen, it was revealed that the confrontation with a focus on religion is commonly more employed than the search for social support and emotion, and social support was more employed than the emotion. In addition, problem-focused coping was more widely used than religious practices or the pursuit of social support and emotion^15^. In the present study there was a small correlation between religion-focused coping with coping with focus on emotion and coping with focus on the social support with coping with focus on emotion. A reasonable degree of correlation was obtained between confrontation with a focus on religion and coping with focus on emotion and coping with a focus on social support and problem-focused coping. It is worth noting that, according to results obtained by authors^13^, positive religious coping strategies and spiritual well-being should be strengthened, and negative religious strategies should be recognized and avoided as they are related to a higher level of anxiety, less use of positive self-affirmations, and less spiritual wellbeing.

The anxiety trait of women with breast cancer undergoing chemotherapy was shown to be inversely proportional to the focus on the problem and a relation directly proportional to the focus on the emotion. Thus, the higher the anxiety trait, the less the problem-focused coping, but it will a greater use of the emotion-focused strategy, which is in accordance with the results obtained by other authors^10^ ^,^ ^16^. Yet another study^17^ ^)^ reveals that the more the patient uses emotion-focused coping, the more likely they will have anxious and depressive symptoms. However, the greater the focus on the problem, the lower the chances of the patients having depressive symptoms.

A study^10^ with patients in the preoperative period verified that there is a significant positive trend between the focus on emotion and the state of anxiety. Thus, the higher the level of anxiety state, the more employed this type of coping will be. In the same study, there was a negative correlation between the state of anxiety and the coping strategy focused on social support and problem solving. Thus, these strategies will be more used according with the lower the level of anxiety state, which is in agreement with the results obtained in the present research. Here it was observed that the higher the state of anxiety, the greater the use of the coping with focus on emotion and there will be less employment of the problem-focused strategies. The anxiety state of women with breast cancer has also been shown to be directly related to the anxiety trait, which means that people with a high anxiety trait will tend to present high levels of anxiety, representing people who respond more intensely to stressor stimuli^8^.

The use of problem-focused coping may be a strong determinant of adaptation to breast cancer^12^. It is of paramount importance that nurses who experience the care of women undergoing chemotherapy should be aware of the coping strategies used, considering individuality, needs, possibilities and beliefs, in search of interdisciplinary strategies or actions that help them cope more effectively with the process experienced by them. Thus, the results of this study are of great relevance to assist nurses to provide adequate care to women with breast cancer undergoing chemotherapy.

As a limitation of this study, we performed a quantitative analysis of the coping used by the patients, which made it impossible to identify other forms of coping not contemplated by the instrument used. In addition, there is shortage of national and international articles that correlate the trait and state of anxiety with the coping strategy used by cancer patients in chemotherapy treatment.

## Conclusion

It was verified that the coping strategy chosen by women with breast cancer undergoing chemotherapeutic treatment is directly related to their state and anxiety traits. Thus, patients who have the low level anxiety trait and state tend to face it using the problem solving strategy. However, when the level is medium to high, there is a trend to employ confrontation with a focus on emotion. The state of anxiety has also been shown to have direct correlation with the anxiety trait.

It is necessary to develop new studies around this topic to increase the options available to professionals who deal with cancer patients, especially those who experience chemotherapy. In addition, as practitioners get to know the correlation between coping and anxiety, they can develop measures to implement and help patients to choose effective coping strategies consequently reducing the level of anxiety of their patients, which will be of great value for the treatment.
